# Inferring Hierarchical Orthologous Groups from Orthologous Gene Pairs

**DOI:** 10.1371/journal.pone.0053786

**Published:** 2013-01-14

**Authors:** Adrian M. Altenhoff, Manuel Gil, Gaston H. Gonnet, Christophe Dessimoz

**Affiliations:** 1 Department of Computer Science, ETH Zurich, Zurich, Switzerland; 2 Swiss Institute of Bioinformatics, Zurich, Switzerland; 3 EMBL-European Bioinformatics Institute, Hinxton, Cambridge, United Kingdom; Université Paris-Sud, France

## Abstract

Hierarchical orthologous groups are defined as sets of genes that have descended from a single common ancestor within a taxonomic range of interest. Identifying such groups is useful in a wide range of contexts, including inference of gene function, study of gene evolution dynamics and comparative genomics. Hierarchical orthologous groups can be derived from reconciled gene/species trees but, this being a computationally costly procedure, many phylogenomic databases work on the basis of pairwise gene comparisons instead (“graph-based” approach). To our knowledge, there is only one published algorithm for graph-based hierarchical group inference, but both its theoretical justification and performance in practice are as of yet largely uncharacterised. We establish a formal correspondence between the orthology graph and hierarchical orthologous groups. Based on that, we devise GETHOGs (“Graph-based Efficient Technique for Hierarchical Orthologous Groups”), a novel algorithm to infer hierarchical groups directly from the orthology graph, thus without needing gene tree inference nor gene/species tree reconciliation. GETHOGs is shown to correctly reconstruct hierarchical orthologous groups when applied to perfect input, and several extensions with stringency parameters are provided to deal with imperfect input data. We demonstrate its competitiveness using both simulated and empirical data. GETHOGs is implemented as a part of the freely-available OMA standalone package (http://omabrowser.org/standalone). Furthermore, hierarchical groups inferred by GETHOGs (“OMA HOGs”) on >1,000 genomes can be interactively queried via the OMA browser (http://omabrowser.org).

## Introduction

Homologous biological sequences–sequences related through common ancestry–can be further classified according to the type of evolutionary event that initiated their divergence from one another. Notably, pairs of genes that descended from their last common ancestor through a speciation are referred to as orthologs, while genes that have diverged from a duplication event are referred to as paralogs [1]. This distinction is useful in a broad range of contexts, such as genome annotation, comparative genomics, and phylogenetic analyses. Accordingly, numerous methods and associated databases have been developed to infer orthology and paralogy (reviewed in [2], [3]).

Orthology between pairs of genes can be quite reliably inferred using various algorithms, such as bidirectional best hit [4], reciprocal smallest distance [5], Inparanoid [6], or OMA pairwise [7] (see [8] for in-depth description and evaluation). Yet, many analyses require relations over more than two genes at a time. But because in general orthology and paralogy are non-transitive relations (i.e. x being orthologous to y and y being orthologous to z does not imply x being orthologous to z), the generalisation of these concepts to sets of genes is not straightforward. As a consequence, several definitions of orthologous groups have been proposed, with considerable differences in terms of evolutionary relations implied [9].

For instance, OrthoMCL identifies groups of orthologs and “close” paralogs using Markov clustering, a procedure to identify sets of genes with high pairwise alignment scores [10]. A more stringent grouping strategy lies in identifying cliques of orthologs, but this comes at the cost of lower coverage in terms of all orthologous relations [7]. Also worth mentioning are criteria which are not directly aiming for orthology, such as groups with a given minimum percentage of sequence identity (e.g. [11], [12]) or minimum percentage of sequence length coverage (e.g. [12]). However, these non-evolutionary criteria can yield groupings which are at odds with the central notion of evolution and function changes along trees.

**Figure 1:**
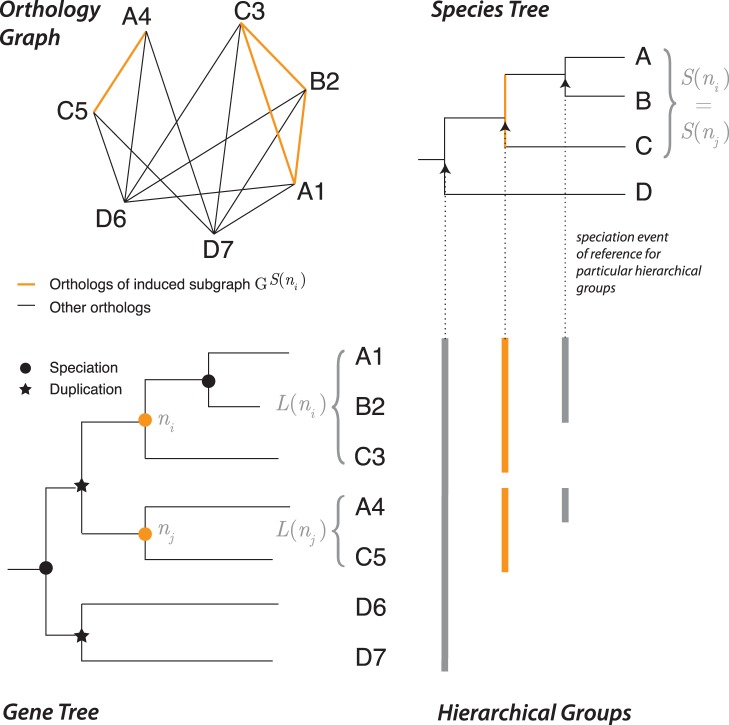
Hierarchical orthologous groups and their relationship to the orthology graph and the underlying gene and species trees. In this example, the hierarchical groups for the taxonomic range 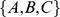 are drawn in orange. By definition, these groups correspond to the sets of leaves attached to the speciation nodes of the gene tree coloured in orange.

One particularly useful gene grouping strategy, sometimes referred to as *hierarchical orthologous groups*, entails grouping genes that have descended from a single common ancestral gene in the last common ancestral species of a given taxonomic range [Figure 1]. This definition has several interesting implications: (i) defining groups in terms of specific taxonomic ranges enables users to fine-tune their analyses to different contexts of investigation–for instance, studying “ubiquitous” genes among all species, but studying lactation genes in terms of the last mammalian common ancestor only; (ii) hierarchical groups have a straightforward interpretation in terms of gene trees: they are clades on these trees; (iii) collectively, hierarchical groups defined with respect to every ancestral species capture all orthologous and paralogous relations [9]. Hierarchical groups are at the heart of the orthology databases EggNOG [13] and OrthoDB [14]. Since 2011, we also provide this type of grouping in the OMA database [15].

Hierarchical groups can be trivially derived from reconciled gene/species trees, such as those obtained by LOFT [16], Ensembl Compara [17], Synergy [18], or PhylomeDB [19]. However, these tree-based approaches are computationally expensive, and indeed, most large phylogenomic databases are “graph-based”, i.e. they infer orthology based on pairwise gene comparison [3]. But most well-established graph-based methods do not attempt to reconstruct hierarchical groups: OrthoMCL groups are a trade-off between orthology and paralogy [20], Inparanoid works for pairs of genomes only [21] and RoundUp computes pairs of orthologs only, i.e. there is no further grouping [22]. To our knowledge, the only graph-based algorithm for hierarchical group inference published to date is COCO-CL [23], which despite its pioneering character is a somewhat ad-hoc approach. Indeed COCO-CL can be shown to return suboptimal results on relatively simple problems, even with perfect input data (example provided in Materials S1).

In this article, we present GETHOGs, which stands for “Graph-based Efficient Technique for Hierarchical Orthologous Groups”. The algorithm is based on correspondences between the orthology graph and the underlying gene phylogeny, correspondences that we prove in two new lemmas. We present an efficient implementation of the algorithm as part of the OMA standalone package. We demonstrate that the resulting algorithm outperforms COCO-CL on simulated and real data. We also show that GETHOGs outperforms the tree reconciliation method LOFT. Lastly, we contrast GETHOGs’s results on real data with predictions of the EggNOG and OrthoDB databases (whose precise algorithms are as yet unpublished).

## Methods

In this section, we first mathematically define hierarchical orthologous groups in terms of gene and species trees, and derive useful notions and properties. We then define the orthology graph, which, crucially, can be inferred without computing gene trees. Next, we describe the correspondence between hierarchical orthologous groups and the orthology graph. The rest of the section details the data and methods used for validating and comparing our new algorithm with existing approaches.

Readers not interested in the technical details can skip this section and proceed directly to the description of GETHOGs (Results Section).

### Labelled Gene Trees, Species Trees, and Hierarchical Orthologous Groups

Let 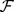 be a forest of rooted gene trees where the internal nodes are labelled either as speciation or duplication nodes. We denote the speciation nodes on these gene trees as 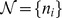. Note that one speciation *event* at the genome level (i.e. on the species tree) corresponds to multiple speciation nodes on the gene trees (one node per ancestral gene in the ancestral species undergoing speciation). The leaves of the trees 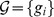 represent present day genes, so that we use the terms leaf and present day gene interchangeably. Furthermore, we require that each gene 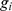 belongs to exactly one gene tree in 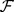. With 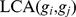, we denote the last common ancestral gene of two present day genes 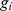 and 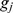. For any present day gene 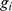, the operator 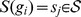 denotes the species that gene belongs to, where 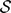 is the set of all species covered by 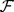. Similarly, we denote by the leaves attached to a speciation node 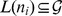 the set of leaves contained in the subtree rooted by the speciation node 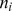 and by 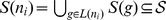 the taxonomic range of 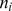, represented by the set of species appearing in the subtree rooted at speciation node 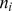 (see Figure 1 for illustration of some of these definitions).

By definition, 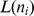
–the set of genes that have descended from the speciation node 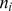
–constitutes one *hierarchical group* for the taxonomic range 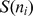. For example, consider an ancestral gene that has duplicated within the vertebrates, but before the mammalian radiation. For the vertebrate taxonomic range, all present day genes that have descended from that ancestral gene are in the same hierarchical group, say 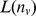, assuming 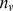 corresponds to the first speciation event among the vertebrates; by contrast, with respect to the mammals, these present day genes are split into two distinct hierarchical groups, say 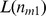 and 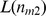, where the speciation nodes 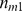 and 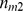 correspond to the first speciation event among the mammals.

Let 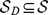 be a chosen *taxonomic range*, such that 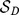 forms a monophyletic group of size 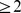 in the species tree. Such a subset of species induces a subset of present day genes 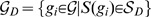. We define 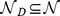 to be the minimal set of speciation nodes whose leaves (i.e. hierarchical groups) collectively include most genes in 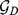 without including genes in species outside 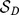. A constructive definition of 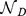 is given by Algorithm 1 (Table 1). Note that in the absence of gene loss, the nodes 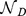 are the ancestral genes in the last common ancestor of 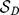, which is a more intuitive way of thinking about this set. From these speciation nodes, we derive i) 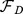 as the forest of subtrees of 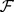 rooted at the speciation nodes 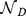; and ii) 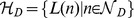, the set of hierarchical orthologous groups induced by speciation nodes 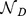.

**Table 1:** Algorithm 1 GroupRoots.

**Input:** Set of rooted gene trees 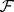 and a taxonomic range 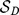
**procedure** TreeGroupRoots( 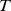 )
**if** 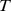 is a leaf **then**
**return ** 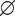
**else**
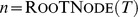
**if** 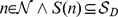 **then**
**return ** 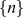
**else**
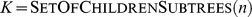
**return ** 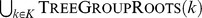
**end if**
**end if**
**end procedure**
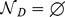
**for all** 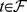 **do**
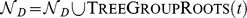
**end for**
**return ** 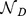
**Output:** Subset of speciation nodes 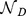

#### Proposition 1

*For all speciation nodes 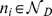, there is no speciation node 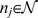 with the two following properties: i) 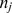 is an ancestral node of 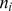; ii) 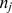 corresponds to a speciation event within the taxonomic range 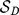.*

#### Proof

We prove this proposition by contradiction. Assume the existence of such speciation nodes 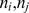. Since 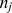 is an ancestral node of 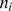, 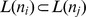. Furthermore, since 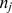 is within the taxonomic range 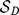, the additional leaves of 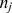 belong to species in 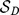 as well (i.e. 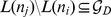). But then, 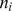 is not part of the minimum set of speciation nodes whose leaves collectively cover most of 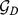 without covering genes in species outside 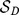, which contradicts our assumption.

#### Proposition 2

*The correspondences between the speciation nodes in 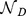, the trees in 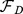, and the hierarchical orthologous groups 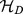 are all one-to-one.*

#### Proof

The one-to-one correspondence between 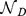 and 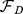 can be established as follows: recall that we require the leaves of the forest 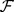 to be distinct; thus, each element in 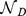 is the root of a distinct gene subtree in 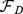. Conversely, each tree in 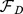 has a distinct speciation node as root. Likewise, the one-to-one correspondence between 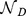 and 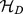 also follows from the requirement that leaves in 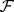 be distinct: this guarantees that the hierarchical orthologous groups associated with each speciation node are distinct; furthermore, by definition, each element in 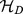 is constructed from a distinct speciation node in 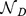. Finally, the correspondence between 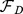 and 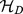 can be viewed as a composition of the two previous one-to-one correspondences, and is therefore one-to-one itself.

#### Proposition 3

*If two present day genes 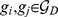 belong to distinct hierarchical orthologous groups in 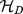, 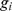 and 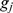 are not orthologous.*

#### Proof

Let 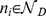 be the speciation node (ancestral to gene 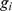) corresponding to hierarchical orthologous group 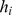, and 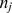 the speciation node (ancestral to gene 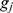) corresponding to 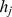. Since 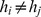 and given Proposition 2, 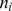 and 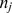 are distinct. We show by contradiction that 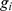 and 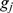 cannot be orthologous. Assume that 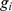 and 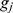 are orthologous. Hence, by Fitch’s definition of orthology, 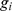 and 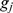 are related through a speciation node 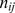, which, since the two genes belong to 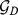, corresponds to a speciation event within the taxonomic range 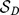. Furthermore, their respective ancestral nodes 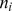 and 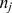 are distinct, which means that 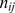 must be ancestral to 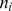 and 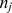. But Proposition 1 states that there is no such speciation node, which contradicts our assumption.

### The Orthology Graph

We define an *orthology graph* to be a graph 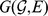 over the present day genes 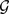 as nodes and with edge set 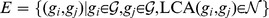, representing pairwise orthology relations between genes as defined by Fitch [1], i.e. they are symmetric, but non-transitive. We further require that every present day gene in 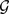 be part of at least one orthologous relation, such that 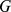 has no singleton. As mentioned in the introduction, pairwise orthologs can be inferred using well-established methods, many of which do not require gene tree reconstruction or gene/species tree reconciliation.

Here, we consider two cases: perfect data, where we assume that the pairwise orthologs have been correctly and exhaustively identified, and “real data”, where these have been imperfectly identified, using OMA pairwise (Sect. “Orthology graph inference”; [7] ).

To restrict the orthology graph to a chosen taxonomic range, we denote by 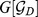 the orthology subgraph induced by the vertex subset 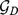, again, without singleton genes. Finally, 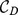 denotes the set of connected components in 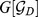. A connected component is defined as a maximal subgraph where there exists a path on the graph between every pair of nodes.

### Correspondence between Hierarchical Orthologous Groups and Orthology Graph

**Figure 2:**
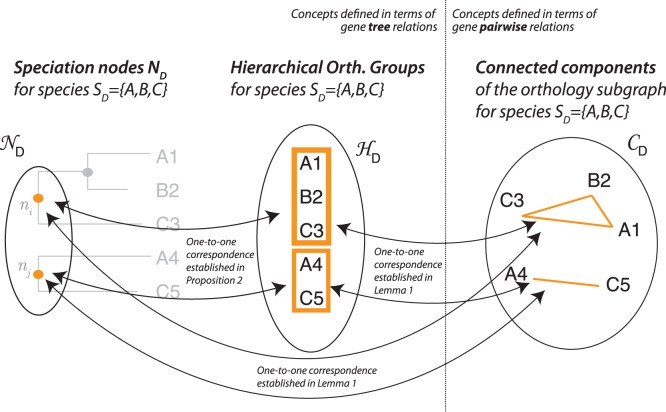
Illustration of Lemma 1: the taxonomic range 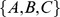 induces a set of speciation node 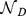 (left) and associated hierarchical orthologous groups 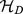 (centre). Likewise, it also induces an orthology subgraph with set of connected component 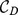 (right). Lemma 1 establishes the one-to-one correspondence between 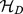 and 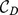 (which we prove by viewing it as composition of the one-to-one correspondences 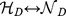 and 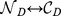).

Our novel algorithm for hierarchical orthologous group inference will use the following two lemmas. The first lemma establishes a correspondence between hierarchical orthologous groups and the orthology graph (illustrated in Figure 2):

#### Lemma 1

*Given a taxonomic range 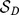, there is a one-to-one correspondence between the hierarchical orthologous groups 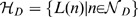 and the connected components 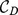 of the orthology subgraph of the taxonomic range in question 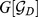.*

#### Proof

As Proposition 2 asserts, the correspondence between the hierarchical orthologous groups 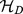 and the speciation nodes 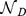 is one-to-one. Thus, it suffice to establish a one-to-one correspondence between 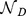 and 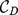. Recall that by definition (and as illustrated in [Figure 1] ), genes in 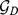 are both the leaves attached to the speciation nodes 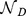 and the nodes in 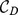. This defines a correspondence between the two sets, which we now demonstrate is one-to-one. First, each element 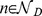 is the root of a tree in 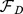, and thus has at least two leaves attached to it; in turn, these leaves belong to at least one 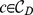. Conversely, any 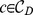 has at least one present day gene; in turn, this gene belongs to at least one 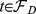 whose root is by definition in 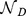. Next, we show that no 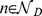 is paired with more than one 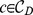. Let 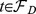 be the tree rooted at 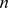. The left and the right subtrees of the speciation node 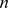 partition the genes in 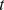 into two sets. By definition, all the genes in one set are orthologous to all the genes in the other set. Therefore, the two sets form a complete bipartite subgraph of 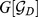 and, hence, lie in one connected component. To conclude the proof, we show by contradiction that no 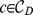 is paired with more than one 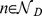. Assume the existence of a connected component 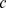 paired with 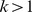 speciation nodes in 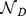. As Proposition 2 establishes, this implies that the connected component 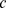 is paired with 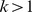 orthologous groups in 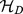. However, Proposition 3 asserts that all pairs of genes belonging to different such groups are non-orthologous, and thus are not connected by an edge in 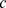. But then, there can be no edge between the 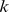 subsets of genes of 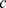 that belong to different groups, which contradicts our assumption that 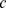 is a connected component.

In the second lemma, we prove that on perfect data, members of a hierarchical group have at most two degrees of separation in the orthology graph. Intuitively, this can be seen by the fact that the deepest split in all considered gene (sub)trees is a speciation node, so every gene in one subtree of this split is orthologous to every gene in the other subtree of that split. Hence, regardless of the relationships within these subtrees, it is always possible to go to another gene within the same subtree by first going to any gene in the other subtree and then coming back.

#### Lemma 2

*Each connected component in 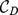 has a diameter of at most 2, i.e. every pair of genes within a hierarchical group is separated by at most 2 edges.*

#### Proof

According to Lemma 1, every 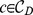 maps onto a 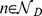. The left and the right subtree of the speciation node 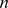 partition the genes into two sets. By definition, all the genes in one set are orthologous to all the genes in the other. Therefore, 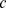 contains a complete bipartite graph known to have at most diameter 2.

We will make use of Lemma 2 to motivate and establish the heuristic FractionReachableInTwoSteps parameter to cope with imperfect input data.

### Methods and Data for Validation and Comparison

#### Simulated genomes

To generate the simulated genomes we used ALF [24], which simulates events at both gene-level (substitution, indels) and genome-level (gene duplication, speciation). For the present work, we simulated 4 independent runs for two different parameter sets: the root genome of each simulation consisted of 200 randomly and independently generated sequences with 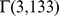-distributed lengths. Although 200 genes is much fewer than in most real genomes, the present work pertains to evolutionary relations *within* homologous families, not among them; as such, the number of starting genes can be viewed as the number of replicates we use to obtain result averages. Sequence evolution was simulated with two M3 codon models [25] with default parameters along a species tree of 30 taxa sampled from a birth-death process with birth rate 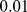 and death rate 0.001. The distance from the root to the leaves was set to 150 PAM. Gene duplication and loss rates both were set to 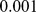 for the first parameter set. For the second parameter set, we set duplication rate to 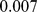 and loss rate to 0.005. In the second parameter set, we additionally allowed temporal rate changes after duplication to model sub- and neo-functionalization of genes as well as gene fusion and fission. The ALF parameter files with all options are provided as supplementary materials (Datasets S1 and S2).

#### Empirical data

We reanalysed the three gene families from a recent manually curated study by Boeckmann et al. [9]. As input for our algorithm, we used the pairwise orthologs from the OMA May 2010 release as orthology graph and the NCBI taxonomy [26] as the species tree.

#### Orthology graph inference

To construct the orthology graph, we used pairwise orthologs inferred by the OMA algorithm [7], which has been shown to be competitive in benchmarking studies [8], [9], [27], [28]. In brief, the OMA algorithm first computes all-against-all sequence alignments using full dynamic programming. From these, potential orthologs (“stable pairs”) are selected based on evolutionary distances and considering inference uncertainty. In a verification step, the algorithm identifies pseudo-orthologs arising through differential gene loss [29]. The resulting “verified pairs” are used to construct the orthology graph for the hierarchical clustering method proposed here.

#### Species-tree inference

With the simulated dataset, we do not assume knowledge of the true species tree. Instead, we estimate it using a least-squares distance approach (*MinSquareTree()* function in Darwin; [30]), using OMA groups as sets of marker genes [7].

#### COCO-CL on COG clusters

COCO-CL requires initial homologous clusters and refines them into a hierarchy by applying a single linkage clustering algorithm on the induced pairwise distance estimates of the cluster’s multiple alignment. As suggested by the authors [23], we built the initial clusters using the COG algorithm [31]. The COG parameters were chosen according to software documentation, i.e. E-value cutoff = 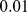 and hit coverage threshold  = 0.5. We applied COCO-CL on both the simulated and real datasets. On simulated data, and in order to assess the COCO-CL gene family refinement procedure independently from the COG clustering step, we also used the true simulated homologous gene families as input clusters. To conform to the definition of hierarchical groups, we fixed COCO-CL’s paralogy threshold 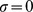, i.e. two sub-clusters sharing genes from the same species have to be related by a duplication. For the analysis on simulated data, we varied the bootstrap threshold between 0 and 0.95. For the analysis on empirical data, we set the bootstrap threshold to the default value (0.75).

#### LOFT

LOFT is a tree-based orthology inference method [16]. It computes Neighbour-Joining gene trees based on pairwise distances using the 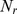 model [32] followed by an evolutionary event-labelling step of the internal nodes based on a species overlap criterion. Similarly to COCO-CL, LOFT requires initial gene families to work on. Again, we use the inferred COG clusters using the parameters as described above on both simulated and real datasets. On the simulated dataset, as additional control, we repeated the analyses using the true and complete homologous gene families as input.

#### Performance metric

Following Boeckmann et al. [9], we measured the performance of a method in terms of the precision and recall of *pairwise* orthology or paralogy. Precision and recall are defined as 
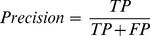
 and 
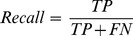
, where 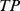 is the number of true positive reported relations, 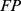 the number of spuriously reported relations and 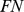 the number of missing predictions. Both precision and recall are bound to the interval 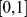, with higher values indicating better performance.

## Results and Discussion

We first present an algorithm which, given a perfect input orthology graph (i.e. all the pairwise orthologs have been correctly and exhaustively identified) and the true (partially or fully resolved) species tree topology, correctly identifies for all taxonomic ranges the corresponding hierarchical orthologous groups. In the second part, we present extensions to cope with imperfect data followed by some remarks about the implementation of the algorithm. We conclude this section by comparing the performance of GETHOGs with existing methods.

### GETHOGs Algorithm

#### Perfect input data

In order to obtain a hierarchy of nested orthologous groups, our approach requires a rooted, at least partially resolved species tree. Our proposed algorithm computes a hierarchy of orthologous groups by recursively identifying the connected components on the orthology subgraphs induced by the species in the lineages at various taxonomic levels (Algorithm 2, Table 2). As Lemma 1 shows, these connected components directly correspond to hierarchical groups. The corollary to this is that other clustering criteria are suboptimal in at least some cases. For example, the COG triangle-based algorithm is too restrictive when we reach the hierarchical level of two species only. At the same time, it can erroneously merge different groups if they are related through speciation events outside the taxonomic range of interest. More stringent clustering approaches (e.g. MCL with typical parameters) will fail in other cases.

**Table 2:** Algorithm 2 GETHOGs.

**Input:** Rooted species tree 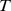 and orthology graph 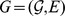
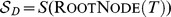
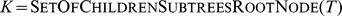
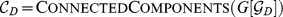
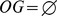
**for all** 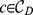 **do**
**for all** 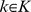 **do**
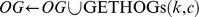
**end for**
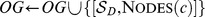
**end for**
**return** 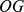
**Output:** Set of tuples of taxonomic range and associated orthologous groups.

Note that, due to the definition of hierarchical groups, genes belonging to different groups at the same taxonomic range have descended from distinct genes in the last common ancestor. As we have formally established in Proposition 3, such genes are in no circumstance orthologous, and are paralogous if the groups are evolutionarily related (homologous).

The runtime complexity of the GETHOGs algorithm on perfect input data is 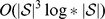, where 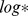 denotes the iterated logarithm function (which grows at a much slower rate than the logarithm function itself). Indeed, algorithm essentially traverses the species tree. In the worst case, the species tree is completely unbalanced. Hence, there are at most 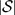 levels of recursion. Within each recursion, we need to compute the following elements: first, computing the induced subgraph requires visiting every edge in the orthology graph. This can be done in 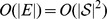, because that the number of genes is bound by 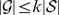, were 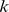 is the number of genes in the largest genome. Second, we need to be able to access the children of the root, which can be done in the worst case, a star tree, in 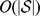. And third, computing the connected components in a graph can be done in 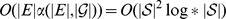, where 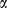 denotes the inverse Ackermann function [33]. Hence, the time complexity of each level is dominated by this last step, which multiplied by the of recursion gives the overall time complexity.

#### Imperfect input data

**Figure 3:**
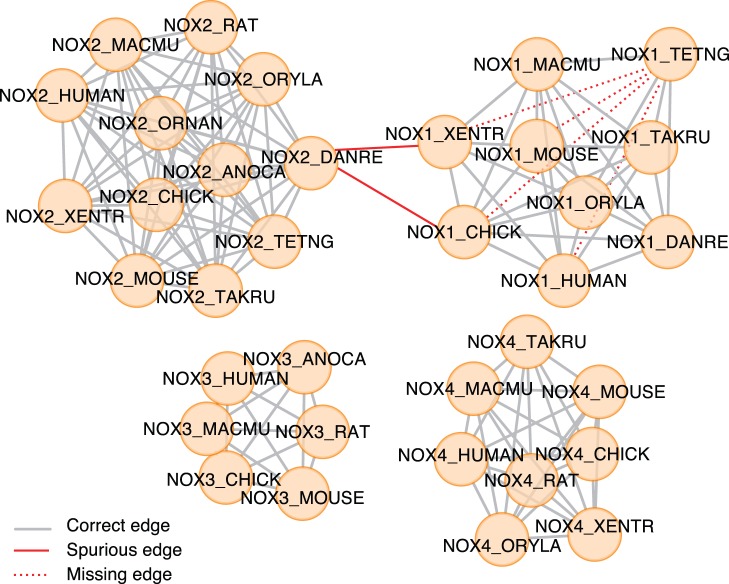
Example of an orthology graph. An example orthology graph from the OMA database where two false positive prediction merges two well-defined orthologous groups. At the level of vertebrates, the *NOX* family forms 4 different orthologous groups. Because of two spurious predictions, the *NOX1* and *NOX2* clusters get weakly connected. The minimum cut algorithm will split them, as there are only two edges to cut.

The two Lemmas described in the “Methods” section are only valid for perfect data. In practice, for all but trivial examples, the input orthology graph can be expected to have missing (false negative) and spurious (false positive) orthology predictions. While missing predictions are typically not a problem–the orthology graph is normally dense enough to provide a path from every group member to every other–additional predictions are more disruptive: false positives result in the erroneous merging of orthologous groups. Hence, using the transitive closure of the pairwise orthology relations would in such situations lead to excessively large clusters. Fortunately, these spuriously merged clusters are often not strongly connected to each other, with only few edges connecting them [Figure 3].

To cope with such errors in the orthology graph, we modify/extend the algorithm GETHOGs (Algorithm 2, Table 2) in the following way: We replace the ConnectedComponents function by DivideGraph (Algorithm 3, Table 3) : this procedure divides the orthology graph using a Minimum-Cut algorithm [34] until all the subgraphs conform sufficiently to the property established in Lemma 2. Minimum-Cut is the well-known computer science problem of cutting a graph into two disjoint components by removing the smallest number of edges (or, in the weighted version, edges with the smallest sum of edge weights). Treating our problem as Minimum-Cut is reasonable in that cutting the graph is needed to undo the effect of spurious edges across groups, while the minimum criterion satisfies the parsimony principle.

**Table 3:** Algorithm 3 DivideGraph.

**Input:** Orthology graph 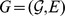 and 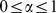
**if** 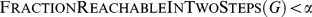 **then**
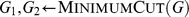
**return** 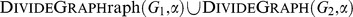
**else**
**return** 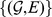
**end if**
**Output:** Set of graphs all satisfying the reachability condition

As for the termination criterion, it is motivated by the property that with correct input, connected components graphs have diameter of at most 2 (Lemma 2). To approximate the diameter, which is expensive to compute, we estimate the average fraction of nodes which are reachable within two steps of each node (Algorithm 4, Table 4). On perfect data, where the diameter is at most 2, this statistic is necessarily equal to 1. On real data, we however allow for values lower than 1, using the stringency parameter 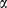. Based on empirical analyses (see below), we have chosen 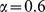 as the default parameter.

**Table 4:** Algorithm 4 FractionReachableInTwoSteps.

**Input:** Orthology graph 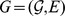 with AdjacencyTable 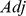
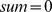
**for** a constant number 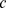 of randomly chosen 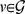 , without replacement **do**
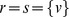
**for** 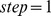 to 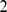 **do**
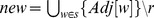
**if** 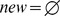 **then**
**break**
**end if**
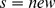
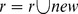
**end for**
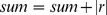
**end for**
**return** 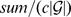
Estimate of average fraction of nodes reachable within 2 steps

Furthermore, it is also possible to use the weighted version of Minimum-Cut. For this purpose, we augment the orthology graph with edge weights corresponding to pairwise alignment scores, and use these weights to guide the Minimum-Cut algorithm. The rationale is that spurious false positives often have relatively low alignment scores. Hence, the spurious edges erroneously connecting two bona fide groups will have low scores and thus be targeted by the weighted Minimum-Cut procedure. But note that while this heuristic has a theoretical motivation based on our findings on perfect data, we do not claim it to be optimal.

We now give an asymptotic runtime analysis of our algorithm. Giving a tight bound on the runtime analysis on imperfect input data is not easy. We therefore make the assumption that gene duplications and losses are distributed uniformly on the gene trees (thus resulting in a mostly balanced gene family tree).

The time complexity of the DivideGraph algorithm depends on that of MinimumCut and FractionReachableInTwoSteps. FractionReachableInTwoSteps runs in order 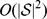. Essentially, we have to traverse the graph in breadth-first order from a constant set of starting nodes. The algorithm by Karger and Stein [35] finds a minimum-cut in 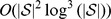. Hence, the time complexity of FractionReachableInTwoSteps is dominated by MinimumCut.

The depth of the recursion DivideGraph depends heavily on the structure of the orthology graph. Obviously, it is limited by the number of nodes, but generally, many fewer iterations are necessary. With the assumption that duplications and losses are uniformly distributed on the gene tree, we can expect the graph to be partitioned in proportions of the total size. Then, 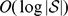 iterations are required, which leads to a time complexity for DivideGraph of 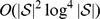.

The resulting overall time complexity for GETHOGs on imperfect data is therefore of order 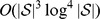.

### Implementation

The source of the described algorithm is freely available for non-commercial uses as part of the OMA standalone package on http://omabrowser.org/standalone. The implementation is written in *Darwin*, an interpreted computer language tailored for bioinformatics applications [30]. An important part for our algorithm is a fast implementation of the *Minimum Cut* algorithm. As a new part of *Darwin*, we added a C implementation of the randomised minimum cut algorithm by Karger and Stein [35].

The Karger-Stein algorithm was implemented for weighted graphs. The algorithm is randomised, that is to say, with certain probability (which can be made arbitrarily small) it may not find the minimum cut, but one slightly larger than the minimum. In practice, we could not find cases where it failed for the default parameters, and even if it would fail, this would mostly alter the order in which we find the groups. This randomization allows us to parallelise the procedure for very large graphs [Materials S1].

### Comparison with Existing Methods

We applied our algorithm to both simulated and real data problems, and compared them to a graph-based and a tree-based hierarchical grouping strategies. We generated two artificial datasets by simulation with ALF [24]: one with moderate gene duplication rate, the other with high duplication rate, rate changes after duplication and gene fusion, and fissions (see Methods). For graph-based COCO-CL, following the authors’ protocol, we inferred initial COG clusters and refined them using different bootstrap parameters ranging from 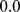 to 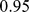 [23]. For tree-based LOFT, also following the authors’ protocol, we inferred one gene tree per COG cluster and inferred duplication nodes by species overlap [16]. For GETHOGs, we used the OMA algorithm [7] to obtain a pairwise orthology input graph. To measure the correctness of the inference in the simulated datasets, we compared the reported induced pairwise orthologous and paralogous relations of the two methods to the true relations obtained from the simulation.

**Figure 4:**
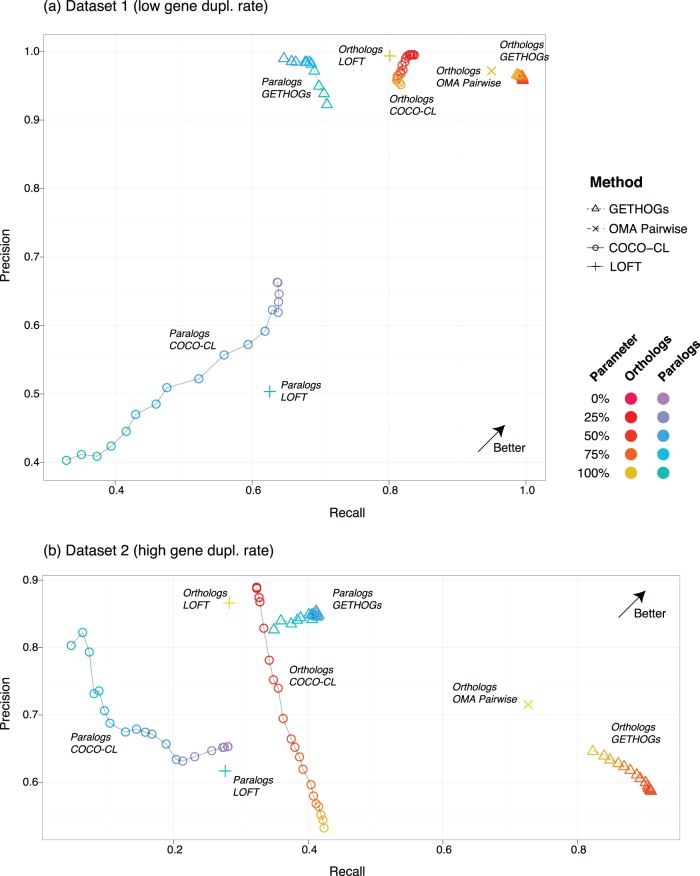
Validation on simulated data: precision-recall plots of COCO-CL, LOFT and the algorithm introduced here (GETHOGs) on two datasets of 30 simulated genomes (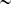
200 genes each). The two datasets show average rates of 4 independent runs of genome simulations with fixed parameters. The difference between the two datasets are essentially different gene duplication rates (see Method section for details). As a point of reference, we also show the performance of pairwise orthologs inferred in OMA (OMA Pairwise). The colour gradient corresponds to various 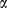 parameter values for GETHOGs and bootstrap value for COCO-CL.

On the dataset with moderate duplication rate, compared to COCO-CL and LOFT, GETHOGs reported considerably more orthologous relations at roughly the same level of precision [Figure 4]. With respect to paralogous relations, GETHOGs strongly outperformed the other methods both in precision and recall. We observed similar trends on the dataset with high duplication rate, except that the precision of GETHOGs orthologs was lower than the precision of COCO-CL and LOFT orthologs. In terms of parameter sensitivity, GETHOGs was little affected by the choice of stringency parameter 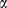 in the first dataset; in the dataset with higher duplication rate, a precision-recall trade-off became apparent, with high 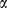 values resulting in moderately higher overall precision and lower overall recall. COCO-CL proved to be more sensitive to parameter choice, with low bootstrap parameter values generally yielding better overall performances.

To analyse the sensitivity to the species phylogeny required by GETHOGs, we ran the algorithm once with the true species tree and once with a species tree inferred from the data (Supplementary Figure 1 in Materials S1). On the first dataset, we observed virtually no difference between GETHOGs with inferred and true species tree, while on the second, more difficult dataset, supplying the true tree led to a modest improvement in precision (Supplementary Figure 1 in Materials S1). This analysis suggests that while GETHOGs can benefit from knowing the true species tree, the method remains competitive when the species tree needs to be inferred.

The surprisingly low recall of LOFT with respect to orthologs and paralogs in the more difficult dataset can be mainly attributed to errors in the gene family inference step, for which LOFT uses the COG algorithm. Indeed, if provided perfect gene family input, the recall for LOFT and COCO-CL increases substantially for both orthologs and paralogs (Supplementary Figure 1 in Materials S1). This suggests that in general the performance of gene/species tree reconciliation methods might strongly depend on the initial family clustering step.

We now turn to the evaluation on empirical biological data. With real data, the true evolutionary relations are mostly unknown. Therefore, we restrict our analysis to a small set of thoroughly studied gene families, which we assume to be free of errors [9]. Again, we compared the predicted relations with the induced relations from these labelled reference gene trees.

This analysis covers three gene families, the “ancestral-type” subfamily of NADPH oxidases (*NOX*1-4), the Popeye domain family (*POP*) and the the eukaryotic V-type ATP synthase beta subunit subfamily (*VATB*). All three families contain at least one lineage specific gene duplication but no horizontal transfer and no major change in their single-domain structure.

**Figure 5:**
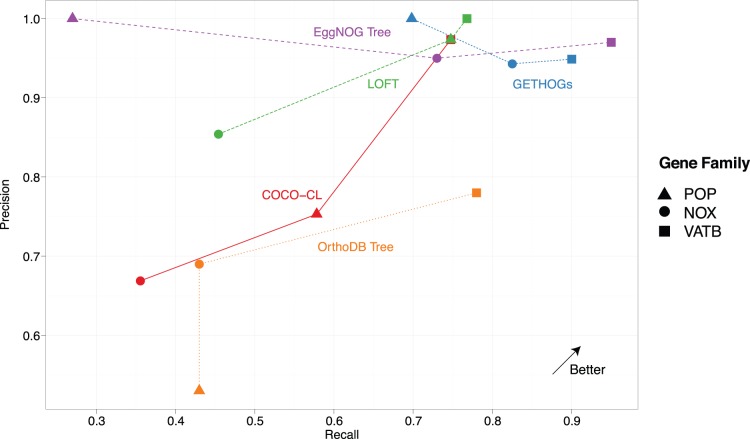
Validation on empirical data: precision-recall plot of our newly proposed *GETHOGs*, *COCO-CL*, *LOFT*, *EggNOG* and *OrthoDB* on orthologous and paralogous gene relationships for the 3 gene families (3,783 relationships in total) analysed in Boeckmann et al. [**9**] **.** Predictions for *GETHOGs* and *COCO-CL* are computed using the default parameters (respectively 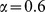 and bootstrap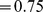). The points for *EggNOG* and *OrthoDB* are from the original analysis (Reference [9],table 2).

In this analysis, we observe that the predictions of GETHOGs largely outperform the ones of OrthoDB and COCO-CL in terms of precision and recall [Figure 5]. Compared with EggNOG and LOFT, the differences are more modest: while EggNOG outperforms our method slightly on the *VATB* gene family, our predictions are considerably better for the *POP* family. As for LOFT, it showed similar performance to GETHOGs for the *POP* and *VATB* families, but did noticeably worse than GETHOGs on *NOX* genes.

Although these 3 families are not sufficient to draw general conclusions, they nevertheless suggest that the good performance of GETHOGs in simulation extends to real data as well. Furthermore, it should be noted that the absence of description of the EggNOG and OrthoDB algorithms, let alone available implementation, precludes their use on custom genomic data.

We finish this section by discussing the limitations of GETHOGs. Most importantly, the method depends on the quality of the input orthology graph. We have established that GETHOGs returns optimal graphs on perfect input data, but we cannot expect perfect input data on real data. Although we have introduced heuristics to cope with errors in the orthology graph, the performance will deteriorate when the input information is not sufficient to discriminate among multiple evolutionary scenarios. We acknowledge that OMA pairwise, which is known to be relatively conservative [8], [28], might not necessarily provide the best input orthologs for GETHOGs; it might for instance be that GETHOGs works better with a more inclusive method, such as Inparanoid (we plan to investigate this question in a later study).

One potential problem with the input graph might be caused by genes encoding multi-domain proteins. Indeed, if the pairwise orthology detection method used to construct the orthology graph does not ensure that orthology between two genes extend over all (or at least most) domains, the resulting graph might strongly violate GETHOGs working assumptions. Note however that the very concept of orthology among genes with different domain composition (and thus non-homologous parts) is ill-defined, as orthology is a subtype of (and thus presupposes) homology. Because of that, many pairwise orthology inference algorithms, including the OMA algorithm we used for all input in this work, require homologous regions between two genes to extend over most of their sequence lengths [7]. Such requirement is sufficient to ensure that there be no orthology inferred between multi-domain genes/proteins with significantly different domain composition.

The other main limitation of GETHOGs lies in the computational cost of processing huge gene families. The currently biggest orthology graph in the OMA database contains 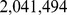 genes and 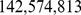 ortholog relations, which is prohibitively expensive for GETHOGs. On very large families, we currently circumvent this problem by starting the GETHOGs recursion at more specific taxonomic levels than the root of all species. Practically, this means that we abstain from resolving the deepest orthology/paralogy relationships in such families. Note however that GETHOGs is able to process most gene families in OMA from the root of the species tree. To give an idea of actual runtimes, computing hierarchical groups on a graph of 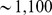 genes and 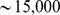 orthologous relations took about 2 minutes on a single desktop computer; processing another graph with 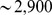 genes and 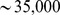 took about 15 minutes.

### Conclusion

We presented GETHOGs, a novel algorithm for reconstructing hierarchical orthologous groups. The approach is based on an orthology graph induced by pairwise orthologous gene relations, and as such requires neither gene tree inference nor gene/species tree reconciliation. The algorithm is motivated by a lemma demonstrating the equivalence of the connected components in the orthology subgraph induced by a taxonomic range and the orthologous groups with respect to the same taxonomic range on perfect data. In order to extend the algorithm to be applicable for real data, we separate weakly connected components by splitting the graph repeatedly at its minimum cut. We stop once the graph is sufficiently densely connected, based on the lemma that the orthology graph should have diameter less than or equal to two.

We applied the algorithm on simulated and real datasets, and compared it to COCO-CL and LOFT, where it finds considerably more orthologs/paralogs at roughly the same precision rate. On real data, we also compared our algorithm to EggNOG and OrthoDB–two databases providing hierarchical orthologous groups–by re-analysing three manually curated gene families from a recent study. Though two the empirical datasets are too small to draw general firm conclusions, the results based on these families indicate that our method is competitive.

Regardless of these promising results, the *raison d*’*être* of GETHOGs lies not so much in resolving once and for all the graph-based hierarchical orthologous group problem as in providing a well-founded and useful starting point to tackle this problem. The theoretical results and implementation provided alongside this study will hopefully foster the development of even better solutions.

## Supporting Information

Materials S1 PDF containing supplementary materials and figures.
(PDF)

Dataset S1 Parameter file used to generate the simulated datasets with low duplication rate with ALF [24].
(DRW)

Dataset S2 Parameter file used to generate the simulated datasets with high duplication rate with ALF [24].
(DRW)
