# Supplementary material for: Inferring Hierarchical Orthologous Groups from Orthologous Gene Pairs
Source: PLoS One. 2013 Jan 14;8(1):e53786. doi: 10.1371/journal.pone.0053786 (PMC3544860; doi:10.1371/journal.pone.0053786)
Supplement: Materials S1 — PDF containing supplementary materials and figures. (PDF) [file pone.0053786.s001.pdf]

Supplementary Material

Inferring Hierarchical Orthologous Groups From Orthologous Gene Pairs

Adrian M. Altenhoff, Manuel Gil, Gaston H. Gonnet and Christophe Dessimoz

Example where the COCO-CL algorithm fails

Assume the following evolutionary scenario,

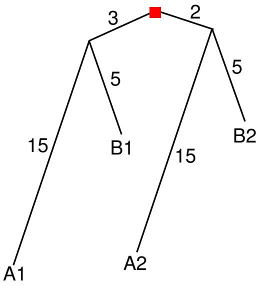

where a duplication occurred at the root of a gene tree (red square) and the genes evolve at different rates after a subsequent speciation (resulting in different branch lengths).

Indexing (A_1, B_1, A_2, B_2) as (1,2,3,4), the distance matrix corresponding to this scenario is

$$D = \begin{pmatrix} 0 & 20 & 35 & 30 \\ 20 & 0 & 25 & 15 \\ 35 & 25 & 0 & 20 \\ 30 & 15 & 20 & 0 \end{pmatrix},$$

and the dissimilarity correlation matrix ($R^* = 1 - r_{ij}, r_{ij}$ being the Pearson correlation coefficient between column vectors D_i and D_j in the distances matrix (see *Methods* section in Jothi *et al.*, 2006)).

$$R^* = \begin{pmatrix} 0.00 & 0.72 & 1.88 & 1.55 \\ 0.72 & 0.00 & 1.63 & 0.68 \\ 1.88 & 1.63 & 0.00 & 0.90 \\ 1.55 & 0.68 & 0.90 & 0.00 \end{pmatrix}.$$

COCO-CL's single linkage clustering algorithm will successively merge genes (((B1,B2), A1), A2), i.e. the last introduced link is A2 vs. (B1,B2,A1). As a result, the wrong groups are inferred:

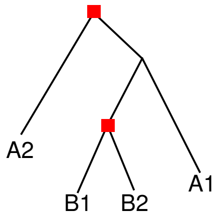

Randomized algorithm allows parallelisation

Here, we briefly describe how the randomized Minimum-Cut algorithm allows for parallelisation of the GETH-OGS algorithm.

Repeatedly computing the Minimum-Cut in a large graph is not easy to parallelise. One obvious way is in case of Karger-Stein's algorithm is to compute the necessary repetitions in different threads. But if the graph is very large, the amount of required memory grows fast. The randomisation allows in a elegant way to do the parallelisation on the level of processes: When loading the graph, one can modify the edge weight for every run randomly in an unbiased way, e.g. $w_i^{'} = w_i + U(0, \sigma)$, where $U(0, \sigma)$ is a uniformly distributed random variable with mean 0 and variance σ. This way, each process will produce a different sequence of Minimum-Cuts given that the graph is large enough and there are many cuts with a similar weights. After a fixed number of iteration, the processes have to synchronize again by combining all the cuts found in the different processes.

References

Jothi, R., Zotenko, E., Tasneem, A., and Przytycka, T. M. (2006). Coco-cl: hierarchical clustering of homology relations based on evolutionary correlations. *Bioinformatics*, **22**(7), 779–788.

Supplementary Figures

(a) Dataset 1 (low dupl. rate)

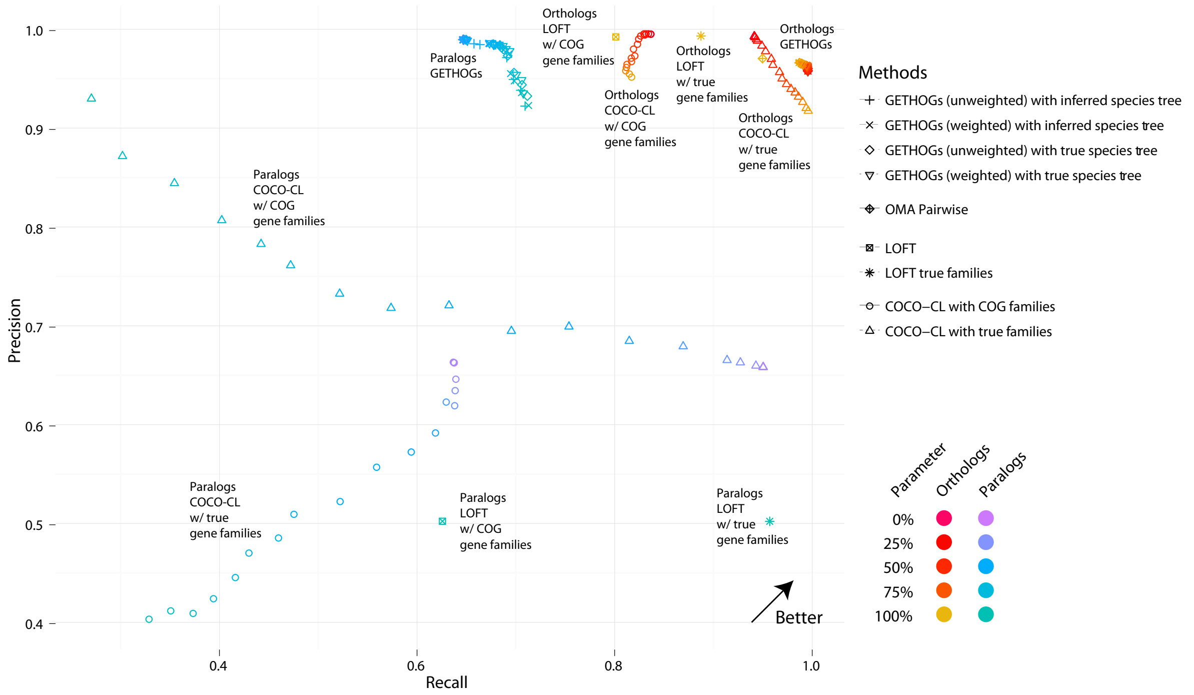

(b) Dataset 2 (high dupl. rate)

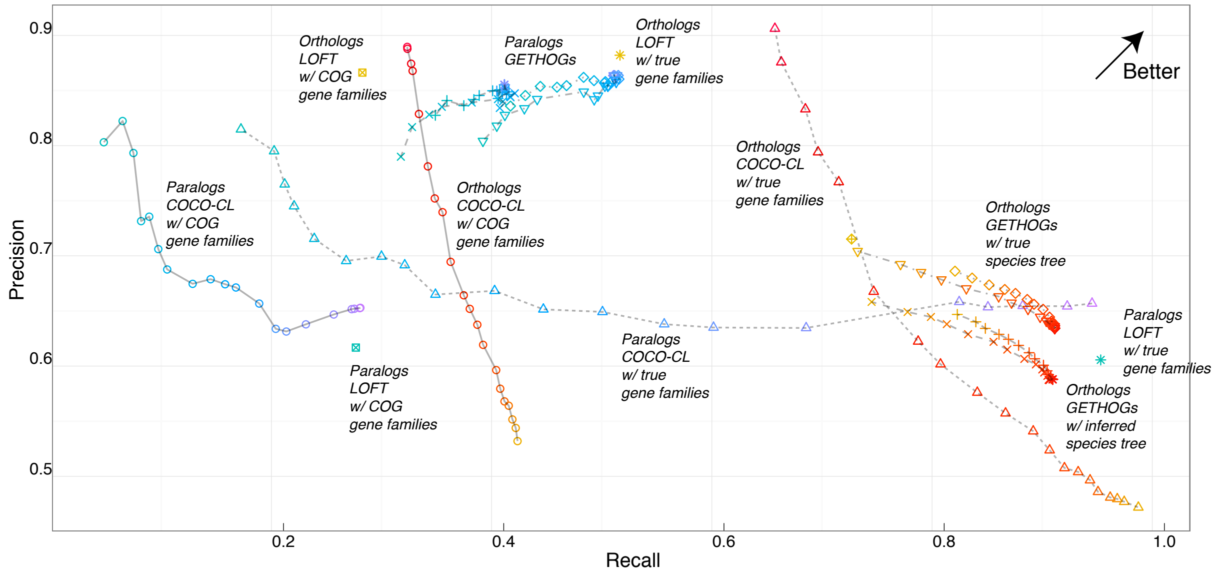

Methods

Supplementary Figure 1: Results of GETHOGs on simulated data with inferred and true species tree with both unweighted and weighted minimum cut algorithm. The difference among the 4 different approaches is unnoticeable (dataset 1) or modest (dataset 2). Results with COCO-CL and LOFT with either COG families, or true gene families. The performance of the two methods strongly depends on the accuracy of the input families.
